# Radiomics integrated with machine and deep learning analysis of T2-weighted and arterial-phase T1-weighted Magnetic Resonance Imaging for non-invasive detection of metastatic axillary lymph nodes in breast cancer

**DOI:** 10.1007/s11547-025-02090-z

**Published:** 2025-09-23

**Authors:** Roberta Fusco, Vincenza Granata, Mauro Mattace Raso, Igino Simonetti, Paolo Vallone, Davide Pupo, Filippo Tovecci, Maria Assunta Daniela Iasevoli, Francesca Maio, Paola Gargiulo, Giuditta Giannotti, Paolo Pariante, Saverio Simonelli, Gerardo Ferrara, Claudio Siani, Raimondo Di Giacomo, Sergio Venanzio Setola, Antonella Petrillo

**Affiliations:** 1 Division of Radiology, Istituto Nazionale Tumori IRCCS Fondazione Pascale – IRCCS di Napoli, Naples, Italy; 2 Division Anatomic Pathology and Cytopathology, Istituto Nazionale Tumori IRCCS Fondazione Pascale – IRCCS di Napoli, Naples, Italy; 3 Division of Breast Surgery, Istituto Nazionale Tumori IRCCS Fondazione Pascale – IRCCS di Napoli, Naples, Italy

**Keywords:** Radiomic analysis, Magnetic resonance imaging, Metastatic axillary lymph nodes

## Abstract

**Purpose:**

To compare the diagnostic performance of radiomic features extracted from T2-weighted and arterial-phase T1-weighted MRI sequences using univariate, machine and deep learning analysis and to assess their effectiveness in predicting axillary lymph node (ALN) metastasis in breast cancer patients.

**Methods:**

We retrospectively analyzed MRI data from 100 breast cancer patients, comprising 52 metastatic and 103 non-metastatic lymph nodes. Radiomic features were extracted from T2-weighted and subtracted arterial-phase T1-weighted images. Feature normalization and selection were performed. Various machine learning classifiers, including logistic regression, gradient boosting, random forest, and neural networks, were trained and evaluated. Diagnostic performance was assessed using metrics such as area under the curve (AUC), sensitivity, specificity, and accuracy.

**Results:**

T2-weighted imaging provided strong performance in multivariate modeling, with the neural network achieving the highest AUC (0.978) and accuracy (91.1%), showing statistically significant differences over models. The stepwise logistic regression model also showed competitive results (AUC = 0.796; accuracy = 73.3%). In contrast, arterial-phase T1-weighted imaging features performed better when analyzed individually, with the best univariate AUC reaching 0.787. When multivariate modeling was applied to arterial-phase features, the best-performing logistic regression model achieved an AUC of 0.853 and accuracy of 77.8%.

**Conclusion:**

Radiomic analysis of T2-weighted MRI, particularly through deep learning models like neural networks, demonstrated the highest overall diagnostic performance for predicting metastatic ALNs. In contrast, arterial-phase T1-weighted features showed better results in univariate analysis. These findings support the integration of radiomic features, especially from T2-weighted sequences, into multivariate models to enhance noninvasive preoperative assessment.

## Introduction

Breast cancer is the most commonly diagnosed cancer among women worldwide, and lymph node status remains a crucial determinant of prognosis and treatment decisions in breast cancer management [1].

The presence of metastatic lymph nodes is associated with worse clinical outcomes, including higher rates of recurrence and lower survival rates [2–4]. Patients with lymph node-positive breast cancer have a significantly lower 5-year survival rate compared to lymph node-negative patients (84% vs. 98%) [5].

Accurate assessment of axillary lymph node (ALN) status remains a crucial aspect of breast cancer staging, as nodal involvement significantly impacts prognosis and therapeutic decision-making. Traditional staging protocols rely on invasive procedures such as sentinel lymph node biopsy (SLNB) and axillary lymph node dissection (ALND), which, although effective, are associated with notable risks including lymphedema, seroma, nerve injury, and impaired arm mobility. Moreover, recent trials [6, 7] have demonstrated that in selected early stage breast cancer patients, ALND may be safely omitted without compromising survival outcomes, challenging the necessity of invasive staging in all patients.

However, these procedures are invasive and associated with potential complications such as lymphedema, nerve damage, and reduced shoulder mobility [8, 9]. While SLNB is less invasive than ALND, it still carries a risk of complications and may not always be necessary, particularly in patients with a low risk of lymph node involvement. Recent clinical trials have shown that in certain cases, omitting ALND in favor of SLNB alone does not negatively impact overall survival [10].

Despite advances in imaging, conventional modalities like ultrasound, mammography, and CT remain limited in sensitivity and specificity, particularly in detecting micrometastases or morphologically normal-appearing nodes. MRI has emerged as valuable tools in preoperative staging, with the potential to detect metastatic involvement in axillary lymph nodes [3, 11–24].

MRI offers superior soft tissue contrast compared to other imaging techniques and has proven effective in assessing both primary breast tumors and regional lymph nodes [4].

Among MRI sequences, T2-weighted imaging and dynamic contrast-enhanced (DCE) T1-weighted imaging have been widely used to evaluate breast lesions and nodal status. T2-weighted images provide high anatomical detail and can reveal changes in tissue architecture and fluid content, while T1-weighted images, especially when enhanced with contrast agents, offer dynamic information about tumor vascularity and perfusion [16]. However, the diagnostic accuracy of these sequences in detecting lymph node metastasis can be enhanced by the application of radiomic analysis.

Radiomics has emerged as a promising tool, capable of extracting quantitative features from standard imaging to capture underlying tissue heterogeneity and phenotypic differences not appreciable by human observers. By analyzing high-dimensional imaging features such as texture, shape, and intensity, radiomics can reveal subtle differences in tissue characteristics that may not be apparent through visual inspection alone [2, 11, 17–30].

Previous studies have demonstrated encouraging results using MRI-based radiomic models for ALN metastasis prediction. Song et al. [5] have demonstrated that radiomic features extracted from DCE-MRI can accurately predict axillary lymph node (ALN) metastasis, showing potential as a noninvasive biomarker to assist in preoperative decision-making. Their study developed a radiomics-based nomogram combining imaging features and clinical factors, achieving an area under the curve (AUC) of 0.874 in predicting ALN metastasis, indicating its clinical utility in reducing unnecessary surgeries and optimizing treatment plans [5].

A recent study by Wang et al. demonstrated the potential of a multi-modality radiomics model combining MRI, mammography, and clinical features for predicting axillary lymph node metastasis in breast cancer patients. Their model achieved high diagnostic accuracy (AUC up to 0.964 in the training set and 0.892 in external validation), outperforming both single-modality models and conventional radiologic assessments. These findings highlight the added value of integrating complementary imaging data and support the clinical relevance of radiomics as a noninvasive tool for axillary staging [31].

A comprehensive systematic review and meta-analysis by Gong et al. [11] evaluated the diagnostic performance of radiomics in predicting axillary lymph node metastasis in breast cancer. By analyzing 25 studies, the authors reported a pooled sensitivity of 82% and specificity of 83%, confirming that radiomics-based models particularly those derived from MRI can provide reliable, noninvasive predictions of nodal involvement. These findings support the growing body of evidence advocating for radiomics as a complementary tool in preoperative axillary staging and underscore its potential role in clinical decision-making [11].

While previous studies and meta-analyses, such as those by Gong et al. [11] and Wang et al. [31], have confirmed the promising role of radiomics in noninvasive axillary staging, several limitations remain, including heterogeneity in imaging protocols, lack of comparative evaluations across different MRI sequences, and limited integration of deep learning strategies.

In this study, we aim to detect metastatic lymph nodes in breast cancer patients by comparing radiomic features extracted from T2-weighted and arterial-phase T1-weighted MRI sequences. A comparative analysis of these modalities may provide insights into the most effective imaging sequence for detecting lymph node metastasis, potentially improving preoperative assessment and leading to more personalized treatment strategies. By leveraging advanced machine learning models to analyze radiomic features, we seek to identify robust imaging biomarkers that can improve the accuracy of metastatic lymph node detection, reduce the need for invasive procedures, and enhance clinical decision-making. Moreover, our study offers a novel contribution by systematically comparing the diagnostic performance of radiomic features extracted from both T2-weighted and arterial-phase T1-weighted MRI sequences integrating both traditional machine learning and deep learning approaches, enhancing methodological robustness.

## Methods

### Dataset characteristics

The study was approved by the local ethics committee (Deliberation No. 1177 dated December 2, 2022, from the National Cancer Institute of Naples Pascale Foundation) and was conducted in compliance with the most recent version of the Declaration of Helsinki and the International Conference on Harmonization of Good Clinical Practice Guidelines. Informed consent was obtained from all subjects.

The inclusion criteria for the study were: (i) female patients aged 18 years or older, diagnosed with invasive breast cancer confirmed by pathological biopsy; (ii) absence of multifocal or bilateral breast cancer; (iii) an M stage of M0; and (iv) no prior history of other tumors. The exclusion criteria for the study were: (i) patients who had undergone preoperative radiotherapy, chemotherapy, or any combination of other treatments; (ii) patients who did not have an MRI examination within two weeks prior to surgery or biopsy; and (iii) absence of a pathological diagnosis of axillary lymph node status.

### MR imaging protocol

High-resolution MR images were obtained using a 1.5 T high-field MRI scanner equipped with a 16-channel breast coil. The imaging protocol included T2-weighted turbo spin echo sequences (STIR TSE) and multiple T1-weighted gradient echo series, performed both before and after the intravenous administration of 0.1 mmol/kg of a positive paramagnetic contrast agent. The contrast medium was delivered at a rate of 2 mL/s, followed by a 40 mL saline flush at the same rate, using an automated injector.

The breast MRI protocol consisted of baseline T1-weighted images followed by six contrast-enhanced T1-weighted acquisitions, each obtained at approximately 60-s intervals. For each patient, image analysis was performed using T2-weighted images and subtracted arterial-phase images from the DCE-MRI. Arterial phase (120 s after contrast agent injection) was analyzed for radiomics analysis. Detailed sequence parameters are provided in Table 1.

**Table 1:** Magnetic resonance imaging scan settings

Settings	T2-weighted STIR TSE	T1-weighted DCE	Units
TR/TE/FA	3800–3090/60–73/150	4.4–5.1/2.0–2.4/15	ms/msdeg/
FOV	250-500X450-500	250-500X450-500	mm^2^
Matrix size	168–384 × 300–350	168–384 × 300–384	pixel
Slice thickness	3	3	mm
Intersection gap	0	0	mm
Pixel spacing	0.83–1.5 × 0.83–1.5	0.89–1 × 0.89–1	mm^2^

Manual segmentation of the lesions was performed by two experienced radiologists, each with 20 to 25 years of expertise in breast MRI. Using the 3D Slicer image computing platform, the radiologists initially segmented the images independently and then reached consensus on the slice-by-slice delineation of each lesion. The segmented masks were used to derive the volume of interest for both arterial phase images of DCE-MRI and TSE STIR T2-weighted sequences.

For each volume of interest, 851 radiomic features were extracted as median values using the PyRadiomics Python package via the 3D Slicer platform. These features adhered to the Imaging Biomarker Standardization Initiative (IBSI) guidelines [32, 33] and included First Order Statistics, shape-based features (2D and 3D), and texture features such as Gray Level Co-occurrence Matrix (GLCM), Gray Level Run Length Matrix (GLRLM), Gray Level Size Zone Matrix (GLSZM), Gray Level Dependence Matrix (GLDM), and Neighboring Gray Tone Difference Matrix (NGTDM). The package also supported wavelet filtering.

### Histological analysis

Following excision through sentinel lymph node biopsy (SLNB) or axillary lymph node dissection (ALND), the lymph nodes are sectioned and stained using hematoxylin and eosin (H&E) to examine the presence of cancer cells. Pathologists assess key features such as tumor size, extranodal extension, and the disruption of normal nodal architecture. Immunohistochemistry (IHC) was also employed to detect micrometastases or isolated tumor cells using cytokeratins markers, which help identify smaller metastatic deposits that may be missed by routine staining.

### Statistical analysis

Inter- and intra-class correlation coefficients (ICC) were computed to evaluate the reproducibility of radiomics features between and within observers. Features with inter- and intra-class ICC values greater than 0.75 were considered to have good reproducibility and were eligible for model construction.

Features were normalized using z-score that standardizes the intensities distribution to have zero-mean and unit-variance, using following equation:

1 $$z_{i} = \frac{{x_{i} - \overline{x}}}{\sigma }$$

where z_i_ is the i-th features’ normalized intensity, x_i_ is the i-th features’ original intensity, and x̅ and σ are, respectively, the mean and the standard deviation of the features within the volume of interest.

Feature selection was performed using a regularized logistic regression model with Elastic Net penalization, which combines L1 (LASSO) and L2 (Ridge) penalties [28]. This approach balances variable selection and shrinkage, reducing the risk of overfitting in the presence of multicollinearity. The optimal value of the regularization parameter (lambda) was determined through tenfold cross-validation, while the mixing parameter alpha was set to 0.5 to equally weight LASSO and Ridge contributions. All features were standardized prior to model fitting, and variables with nonzero coefficients at the optimal lambda were retained for further analysis.

Receiver operating characteristic (ROC) analysis was performed for robust features, with the area under the ROC curve (AUC), sensitivity (SENS), specificity (SPEC), positive predictive value (PPV), negative predictive value (NPV), and accuracy (ACC) calculated.

The classifiers included in the analysis were Logistic Regression (Logit), Random Forest (RF), LASSO, Gradient Boosting Machine (GBM), Neural Network (NN), and Classification and Regression Trees (CART). The dataset was split into a training set (70%) and a test set (30%). Cross-validation (tenfold) was performed on the training set to tune the models and evaluate performance metrics. The best-performing model was selected based on the highest AUC and accuracy scores obtained from the cross-validation on the training set. The performance of the top classifier was then assessed on the test dataset.

ROC curves were plotted for each model, and pairwise comparisons of the ROC curves were performed using DeLong’s test.

Statistical analyses were carried out using R with the caret, pROC, and glmnet packages.

## Results

We retrospectively enrolled 100 breast cancer patients (age 50.6 ± 11 years) and extracted radiomic features from 155 nodal regions (52 metastatic lymph nodes and 103 metastasis-negative lymph nodes) on MRI using T2-weighted and arterial-phase T1-weighted images. The cases were randomly divided into two groups: a training cohort (n = 109, 70%) and a testing cohort (n = 46, 30%). The training set was used to develop and validate the machine learning models, with parameter tuning performed through tenfold cross-validation to ensure optimal performance.

**Fig. 1:**
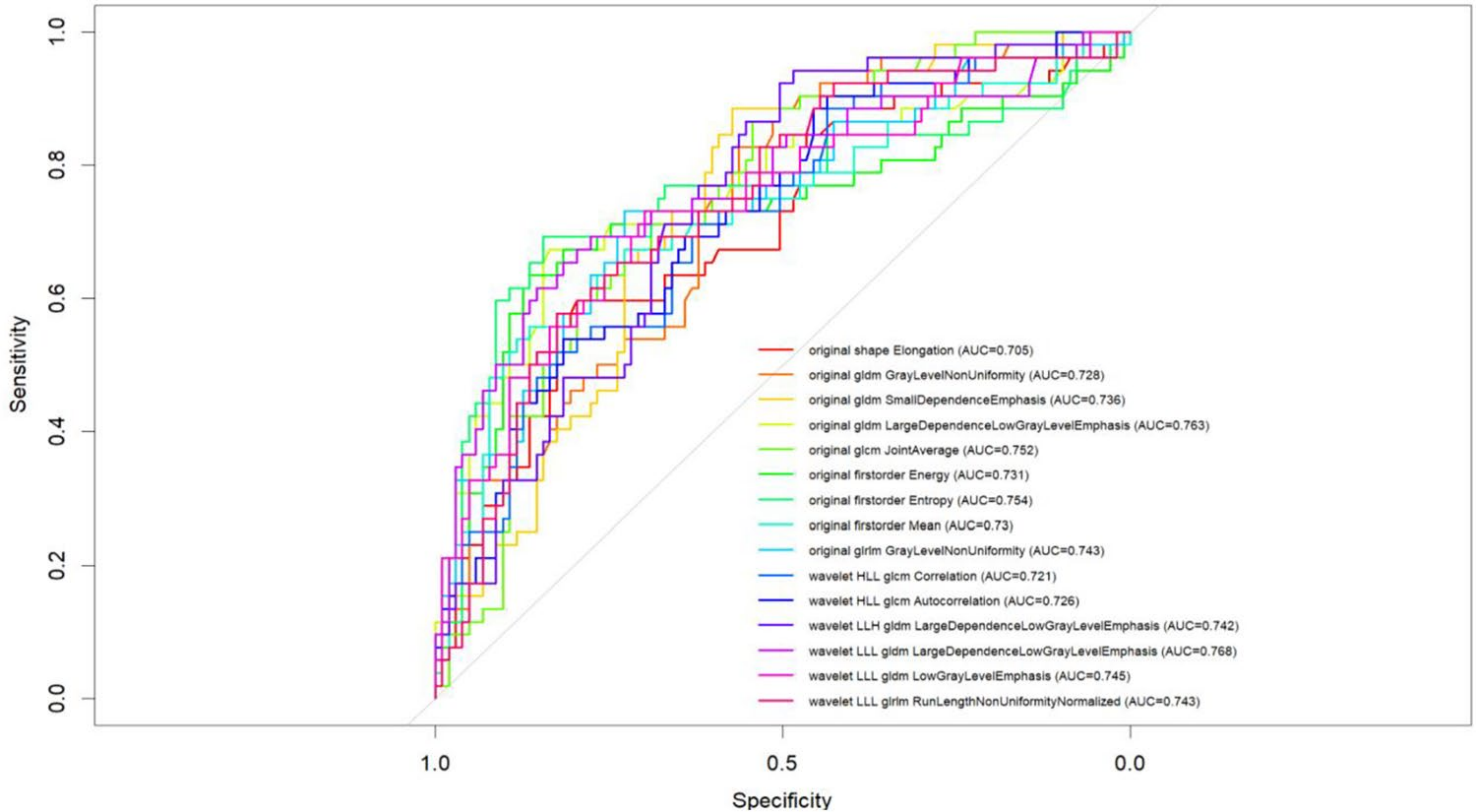
ROC for significant and robust features extracted by T2 weighted images

A total of 70 robust radiomic features were selected from T2-weighted images using Elastic Net regularized logistic regression. Among these 70 features, 15 demonstrated good discriminative performance, with area under the ROC curve (AUC) values ranging from 0.705 to 0.768. The feature LargeDependenceLowGrayLevelEmphasis (from the wavelet LLL gldm class) achieved the highest AUC (0.768), followed by original firstorder Entropy (AUC = 0.754) and original glcm JointAverage (AUC = 0.752). Sensitivity and specificity values varied across features, reflecting a balance between true positive detection and false positive reduction (Table 2 and Fig. 1).

**Table 2:** Performance for features extracted by T2 weighted images

	Feature	AUC	Cutoff	Sensitivity	Specificity	PPV	NPV	Accuracy
1	original shape Elongation	0.705	0.091	0.596	0.796	0.596	0.796	0.729
2	original gldm GrayLevelNonUniformity	0.728	− 0.422	0.827	0.563	0.489	0.866	0.652
3	original gldm SmallDependenceEmphasis	0.736	− 0.229	0.885	0.573	0.511	0.908	0.677
4	original gldm LargeDependenceLowGrayLevelEmphasis	0.763	0.184	0.673	0.835	0.673	0.835	0.781
5	original glcm JointAverage	0.752	0.167	0.865	0.544	0.489	0.889	0.652
6	original firstorder Energy	0.731	0.607	0.635	0.864	0.702	0.824	0.787
7	original firstorder Entropy	0.754	− 0.111	0.692	0.845	0.692	0.845	0.794
8	original firstorder Mean	0.730	− 0.726	0.538	0.883	0.700	0.791	0.768
9	original glrlm GrayLevelNonUniformity	0.743	− 0.212	0.731	0.728	0.576	0.843	0.729
10	wavelet HLL glcm Correlation	0.721	− 0.612	0.519	0.825	0.600	0.773	0.723
11	wavelet HLL glcm Autocorrelation	0.726	− 0.590	0.538	0.816	0.596	0.778	0.723
12	wavelet LLH gldm LargeDependenceLowGrayLevelEmphasis	0.742	0.359	0.923	0.505	0.485	0.929	0.645
13	wavelet LLL gldm LargeDependenceLowGrayLevelEmphasis	0.768	0.309	0.615	0.854	0.681	0.815	0.774
14	wavelet LLL gldm LowGrayLevelEmphasis	0.745	− 0.049	0.731	0.699	0.551	0.837	0.710
15	wavelet LLL glrlm RunLengthNonUniformityNormalized	0.743	− 0.570	0.577	0.825	0.625	0.794	0.742

A stepwise logistic regression model was built using features selected from LASSO-based dimensionality reduction. The final model included seven predictors: original.gldm.SmallDependenceEmphasis; original.shape.Elongation; wavelet.LLH.gldm.LargeDependenceLowGrayLevelEmphasis; wavelet.HLL.glcm.Correlation; wavelet.LLL.glrlm.RunLengthNonUniformityNormalized; original.firstorder.Entropy and wavelet.LLL.gldm.LowGrayLevelEmphasis.

The estimated regression equation was:

$$\begin{aligned} & {\text{logit}}\left( {P\left( {{\text{Metastatic}}\_{\text{LymphNodes}}} \right)} \right) \\ & \quad - 1.292 + 0.997 \times {\text{Elongation}} + 1.006 \times {\text{Entropy}} + 0.556 \\ & \quad \times {\text{RunLengthNonUniformityNormalized}} - 0.624 \\ & \quad \times {\text{LargeDependenceLowGrayLevelEmphasis}} - 0.877 \\ & \quad \times {\text{LowGrayLevelEmphasis}} - 1.196 \times {\text{Correlation}} - 1.764 \\ & \quad \times {\text{SmallDependenceEmphasis}} \\ \end{aligned}$$

Among these, SmallDependenceEmphasis, Elongation, and Correlation were statistically significant predictors (*p* < 0.05). Specifically, higher Elongation and Entropy values were associated with an increased likelihood of a positive outcome (metastatic lymph node), while higher SmallDependenceEmphasis and Correlation were negatively associated with it. The model demonstrated a good fit with a residual deviance of 85.4 on 102 degrees of freedom and an AIC of 101.4. These results suggest that both first-order and texture-based radiomic features contribute meaningfully to the classification task. The stepwise logistic regression model achieved an AUC of 0.796, with an accuracy of 73.3%, sensitivity of 53.3%, specificity of 83.3%, and a negative predictive value (NPV) of 78.1% on the test set.

**Fig. 2:**
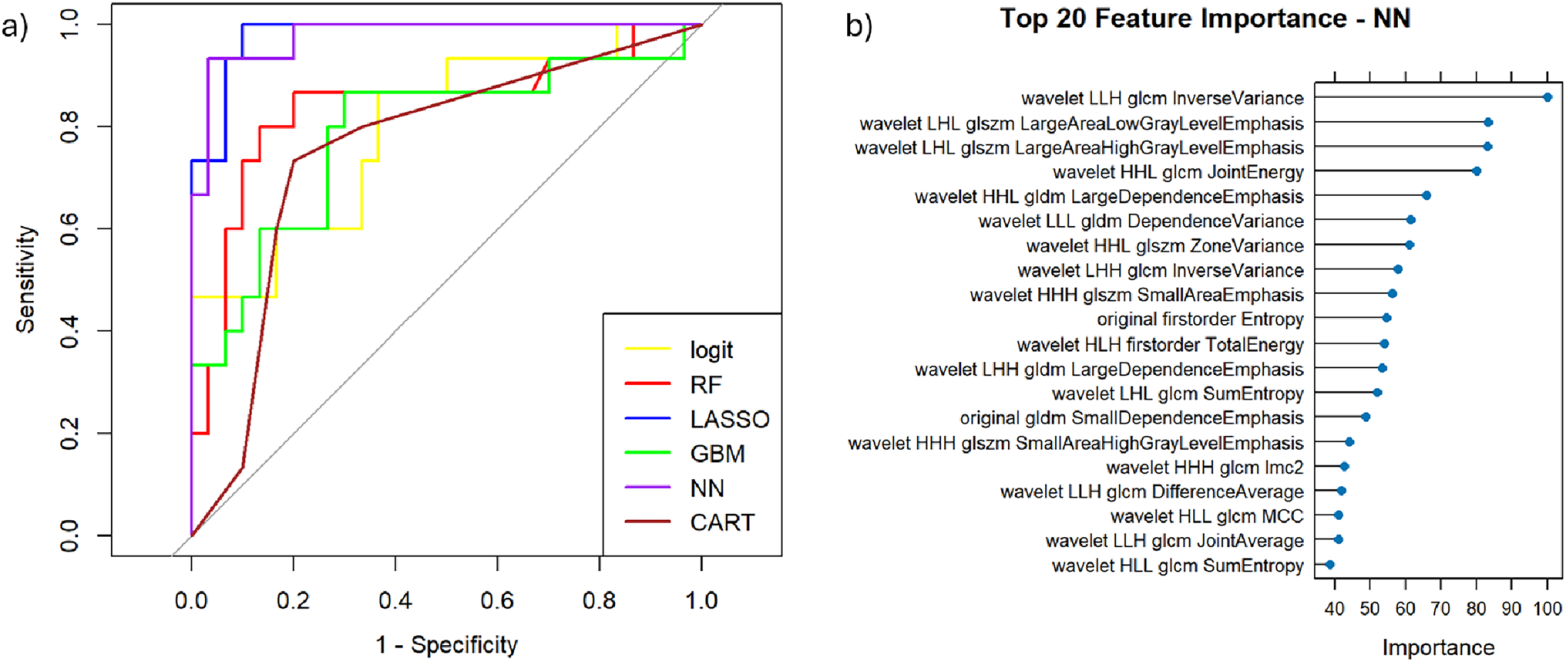
ROC curves for machine learning approaches trained and tested with Radiomics features extracted from T2-weighted images (**a**) and the top 20 features for variable importance obtained by the best NN classifier (**b**)

A comparison of machine learning classifiers trained and tested on the radiomic features extracted by T2-weighted revealed variable performance (Table 3, Fig. 2a).

**Table 3:** Performance for machine learning approaches trained and tested with radiomics features extracted from T2-weighted images

Model	AUC	Accuracy	Sensitivity	Specificity	PPV	NPV
CART	0.756	0.756	0.6	0.833	0.643	0.806
GBM	0.787	0.756	0.533	0.867	0.667	0.788
logit	0.796	0.733	0.533	0.833	0.615	0.781
RF	0.839	0.80	0.60	0.90	0.75	0.818
NN	0.978	0.911	0.80	0.967	0.923	0.906
LASSO	0.980	0.867	0.733	0.933	0.846	0.875

The LASSO and Neural Network (NN) models had the highest AUCs, with values of 0.980 and 0.978, respectively. The Random Forest (RF) model had an AUC of 0.839, and the other models showed lower performance.

DeLong’s test for pairwise comparison of ROC curves revealed the following *p* values: NN vs LASSO: 0.9116 (no significant difference); NN vs GBM: 0.0120 (significant difference); NN vs RF: 0.0503 (marginally significant difference); NN vs CART: 0.0031 (significant difference); NN vs LOGIT: 0.0089 (significant difference). These results indicate that while the NN model showed superior performance with an accuracy of 91.1% compared to other models.

The top 20 most influential features identified (Fig. 2b) by the neural network model are predominantly texture-based radiomic descriptors derived from wavelet-transformed images. The most important feature was wavelet.LLH.glcm.InverseVariance, indicating that local homogeneity in specific frequency-decomposed images played a critical role in classification. Other high-ranking features included LargeAreaLowGrayLevelEmphasis and LargeAreaHighGrayLevelEmphasis from the GLSZM, suggesting that the spatial distribution of low- and high-intensity regions significantly contributed to model predictions. First-order statistics such as Entropy and TotalEnergy were also relevant, highlighting the role of overall image intensity distribution.

**Fig. 3:**
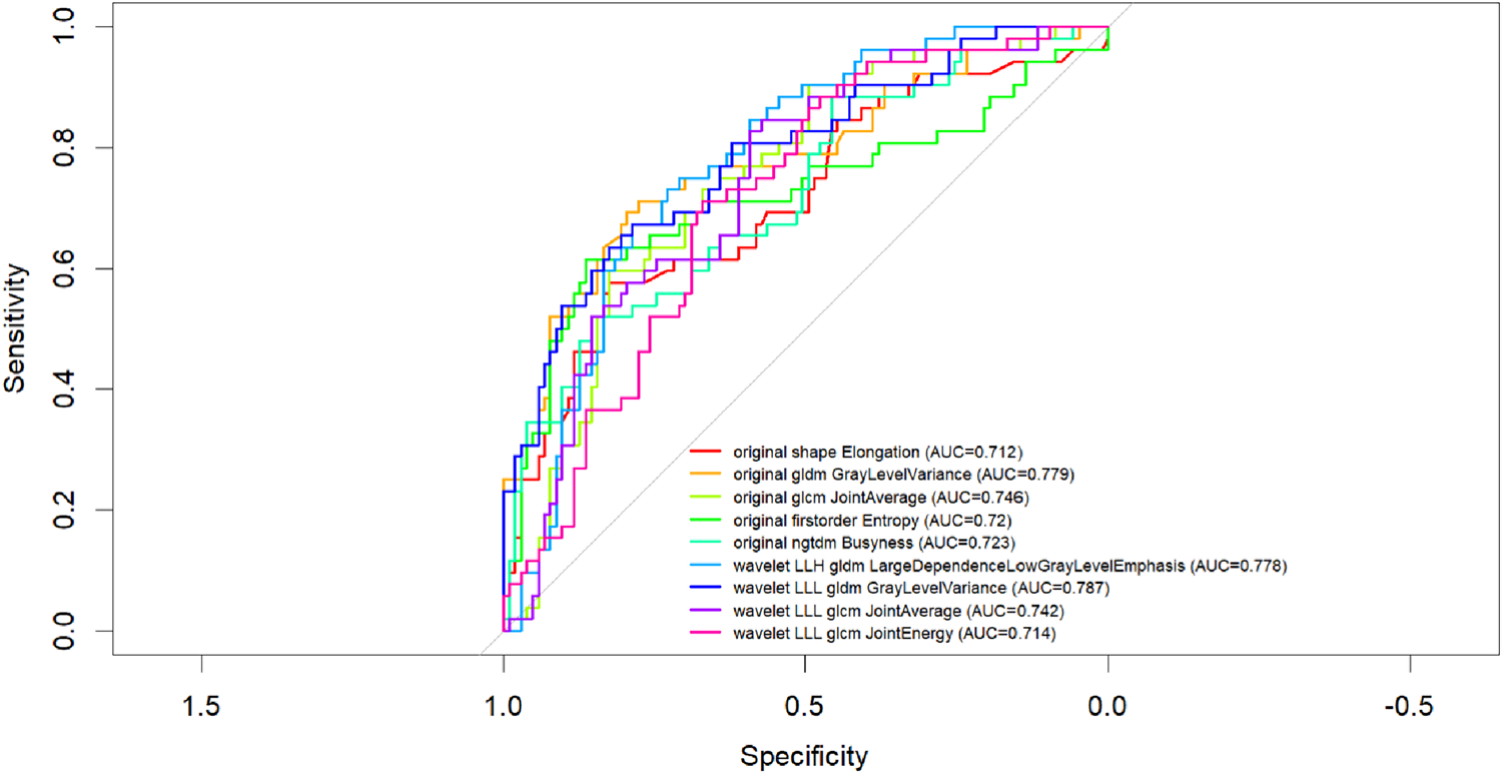
ROC for significant and robust features extracted by arterial-phase T1-weighted images

Thirty-two robust radiomic features were identified from the contrast-enhanced arterial-phase T1-weighted images using Elastic Net regularization. Among these, 9 features exhibited fair to good discriminative ability, with AUC values ranging from 0.712 to 0.787. The highest performance was achieved by GrayLevelVariance (wavelet LLL gldm), with an AUC of 0.787, followed by LargeDependenceLowGrayLevelEmphasis (wavelet LLH gldm, AUC = 0.778) and GrayLevelVariance (original gldm, AUC = 0.779). Sensitivity and specificity values varied across features, with several achieving a balanced trade-off, such as Entropy (original first order) and JointAverage (original gldm) (Table 4, Fig. 3).

**Table 4:** Performance for features extracted by arterial-phase T1-weighted images

	Feature	AUC	Cutoff	Sensitivity	Specificity	PPV	NPV	Accuracy
1	original shape Elongation	0.712	0.067	0.577	0.825	0.625	0.794	0.742
2	original gldm GrayLevelVariance	0.779	− 0.078	0.692	0.796	0.632	0.837	0.761
3	original glcm JointAverage	0.746	− 0.460	0.596	0.825	0.633	0.802	0.748
4	original firstorder Entropy	0.720	0.056	0.615	0.864	0.696	0.817	0.781
5	original ngtdm Busyness	0.723	− 0.409	0.519	0.854	0.643	0.779	0.742
6	wavelet LLH gldm LargeDependenceLowGrayLevelEmphasis	0.778	0.096	0.673	0.786	0.614	0.827	0.748
7	wavelet LLL gldm GrayLevelVariance	0.787	− 0.066	0.635	0.825	0.647	0.817	0.761
8	wavelet LLL glcm JointAverage	0.742	0.121	0.827	0.592	0.506	0.871	0.671
9	wavelet LLL glcm JointEnergy	0.714	− 0.291	0.712	0.670	0.521	0.821	0.684

A stepwise logistic regression model was built using features selected from LASSO-based dimensionality reduction. The final model included seven predictors: wavelet.LLL.gldm.GrayLevelVariance, wavelet.HLH.glcm.InverseVariance, original.shape.Flatness, wavelet.LLH.glszm.SmallAreaEmphasis, wavelet.LHH.ngtdm.Busyness, wavelet.LHH.glszm.LargeAreaLowGrayLevelEmphasis, and wavelet.HLL.gldm.HighGrayLevelEmphasis.

The estimated regression equation was:

$$\begin{aligned} & {\text{logit}}\left( {P\left( {{\text{Metastatic}}\;{\text{LymphNodes}}} \right)} \right) \\ & \quad = - 1.323 + 2.236 \cdot {\text{GrayLevelVariance}} - 1.210 \cdot {\text{InverseVariance}} + 1.458 \\ & \quad \cdot {\text{Flatness}} + 1.431 \cdot {\text{SmallAreaEmphasis}} - 1.360 \cdot {\text{Busyness}} + 0.809 \\ & \quad \cdot {\text{LargeAreaLowGrayLevelEmphasis}} + 0.731 \cdot {\text{HighGrayLevelEmphasis}} \\ \end{aligned}$$

Model performance on the test set showed good discrimination, with an AUC of 0.853, accuracy of 0.778, sensitivity of 0.933, and specificity of 0.700. The positive predictive value (PPV) was 0.609, and the negative predictive value (NPV) reached 0.955, indicating strong ability to rule out false negatives.

**Fig. 4:**
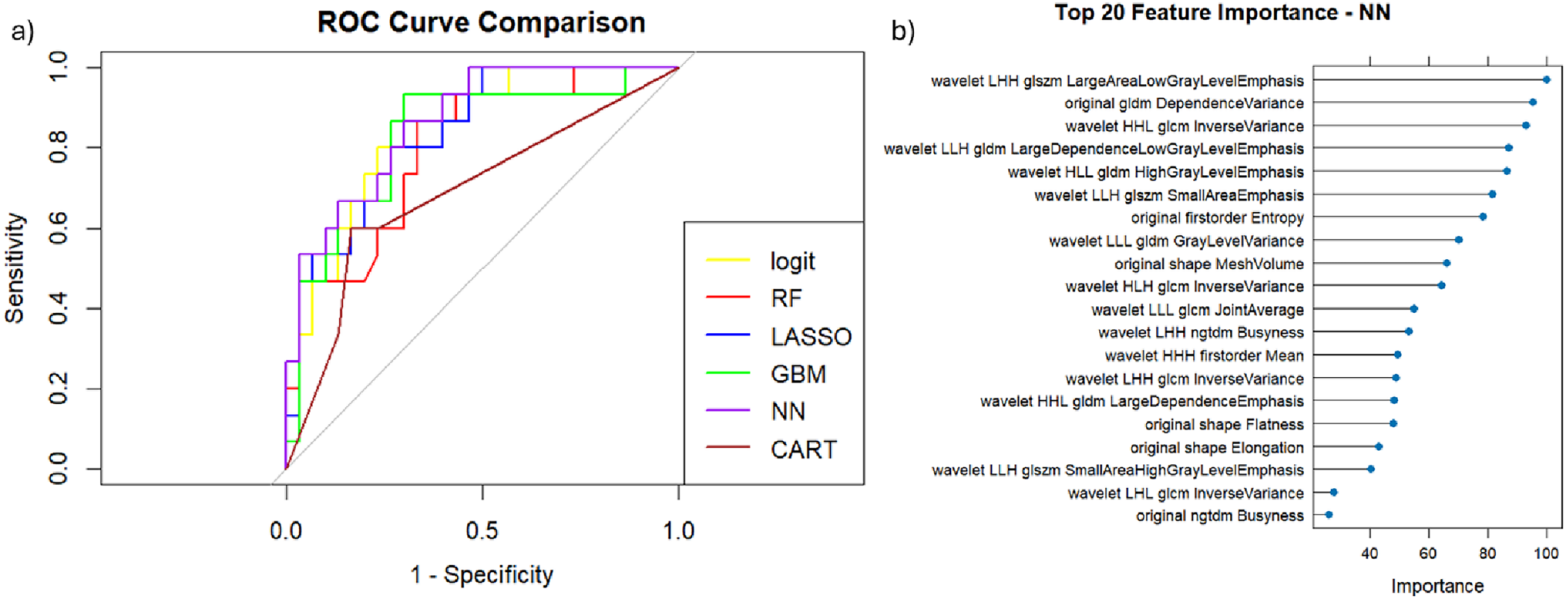
ROC curves for machine learning approaches trained and tested with Radiomics features extracted by arterial-phase T1-weighted images (**a**) and the top 20 features for variable importance obtained by the best NN classifier (**b**)

Among all classifiers (Table 5 and Fig. 4a), NN achieved the highest AUC (0.864) and accuracy (0.800), along with a favorable balance between sensitivity (0.600) and specificity (0.900). Gradient boosting (GBM) and LASSO regression both reached high AUC values (0.831 and 0.833, respectively), although with slightly lower sensitivity. The random forest (RF) model achieved a high specificity (0.933) but lower sensitivity (0.467), indicating a conservative tendency in detecting positive cases. The decision tree (CART) model had the lowest AUC (0.691) but still maintained reasonable accuracy and class balance. Overall, the neural network and stepwise logistic regression emerged as the most effective models in terms of diagnostic accuracy. The NN model showed a significant difference in AUC when compared to the LASSO regression model (*p* = 0.027) and the CART model (*p* = 0.002), indicating a superior performance in both cases. In contrast, no statistically significant difference in AUC was observed between the NN and the GBM (*p* = 0.418), RF (*p* = 0.052), or stepwise logistic regression (*p* = 0.693) models.

**Table 5:** Performance for machine learning approaches trained and tested with Radiomics features extracted by arterial-phase T1-weighted images

Model	AUC	Accuracy	Sensitivity	Specificity	PPV	NPV
CART	0.691	0.756	0.6	0.833	0.643	0.806
RF	0.799	0.778	0.467	0.933	0.778	0.778
GBM	0.831	0.778	0.6	0.867	0.692	0.812
LASSO	0.833	0.756	0.533	0.867	0.667	0.788
logit	0.853	0.756	0.6	0.833	0.643	0.806
NN	0.864	0.80	0.60	0.900	0.75	0.818

The feature importance analysis (Fig. 4b) of the neural network model revealed that texture-related and wavelet-transformed features played a prominent role in classification performance. The most influential variable was wavelet.LHH.glszm.LargeAreaLowGrayLevelEmphasis, followed by original.gldm.DependenceVariance and wavelet.HHL.glcm.InverseVariance. Notably, several features derived from wavelet decompositions, including those associated with gray-level emphasis and spatial variability (e.g., LargeDependenceLowGrayLevelEmphasis, HighGrayLevelEmphasis), were consistently ranked among the top contributors. Additionally, shape descriptors such as Flatness and Elongation, as well as Busyness from the NGTDM category, appeared in the top 20, albeit with relatively lower importance scores.

## Discussion

The results of our study confirm that radiomic features extracted from both T2-weighted and arterial-phase T1-weighted MRI sequences can serve as reliable imaging biomarkers for detecting metastatic axillary lymph nodes (ALNs) in breast cancer patients. In particular, when analyzed independently, several radiomic features demonstrated moderate-to-good discriminatory power. For example, the best-performing single feature extracted from T2-weighted images was wavelet.LLL.gldm.LargeDependenceLowGrayLevelEmphasis, with an AUC of 0.768, followed by original firstorder Entropy (AUC = 0.754) and original glcm JointAverage (AUC = 0.752). These features, which reflect tissue complexity and heterogeneity, are consistent with previous findings that highlight entropy and texture-based metrics as strong indicators of malignancy [4, 11, 17].

When examining features from arterial-phase T1-weighted sequences, diagnostic performance improved further. The top-performing features included wavelet.LLL.gldm.GrayLevelVariance (AUC = 0.787), original gldm GrayLevelVariance (AUC = 0.779), and wavelet.LLH.gldm.LargeDependenceLowGrayLevelEmphasis (AUC = 0.778). These results suggest that arterial-phase imaging, particularly after contrast enhancement, may better capture structural and perfusion-related differences between metastatic and non-metastatic nodes.

Our findings underscore distinct diagnostic contributions from radiomic features extracted from T2-weighted and arterial-phase T1-weighted (subtracted) MRI sequences in the classification of axillary lymph node (ALN) metastases. In the analysis of T2-weighted images, a stepwise logistic regression model based on features selected via LASSO identified seven predictors, including SmallDependenceEmphasis, Elongation, and Correlation, among others. This model achieved an AUC of 0.796, with an accuracy of 73.3%, sensitivity of 53.3%, specificity of 83.3%, and an NPV of 78.1% on the test set. Although performance metrics indicated moderate diagnostic ability, especially in correctly excluding negative cases, sensitivity remained relatively limited, which may reduce clinical utility in detecting all metastatic nodes.

In contrast, modeling using arterial-phase T1-weighted subtracted images produced superior results. A multivariate stepwise logistic regression model incorporating GrayLevelVariance, InverseVariance, Flatness, SmallAreaEmphasis, Busyness, LargeAreaLowGrayLevelEmphasis, and HighGrayLevelEmphasis demonstrated significantly enhanced classification performance: AUC of 0.853, with an accuracy of 77.8%, sensitivity of 93.3%, specificity of 70.0%, and NPV of 95.5%, demonstrating a markedly stronger ability to correctly identify and exclude metastatic involvement.

Furthermore, when comparing machine learning classifiers trained on features from each imaging sequence, the neural network (NN) model achieved its best performance using radiomic features extracted from T2-weighted images, with an AUC of 0.978 and accuracy of 91.1%, demonstrating a strong balance between sensitivity (80.0%) and specificity (96.7%). In contrast, when applied to features from arterial-phase T1-weighted images, the NN model achieved a slightly lower AUC of 0.864 and accuracy of 80.0%.

These findings emphasize that T2-weighted radiomic features provided the strongest input for predictive modeling, particularly when leveraged through deep learning approaches such as neural networks.

Taken together, the results demonstrate that while both T2-weighted and arterial-phase T1-weighted subtracted sequences contribute meaningful radiomic features for axillary lymph node (ALN) assessment, T2-weighted imaging features showed greater predictive power when used in advanced machine learning models, particularly the neural network (NN). The NN model achieved its highest performance with T2-weighted features, yielding an AUC of 0.978 and accuracy of 91.1%, confirming the strong diagnostic potential of non-contrast MRI sequences in this context. This suggests that T2-weighted radiomics may be sufficient or even preferable for ALN classification, particularly in situations where contrast use is limited or contraindicated.

Conversely, when analyzing radiomic features in isolation (i.e., without multivariate modeling or deep learning), arterial-phase T1-weighted features demonstrated superior individual performance. For example, GrayLevelVariance from wavelet-transformed T1 images achieved an AUC of 0.787, outperforming the top single features from T2-weighted images. These findings indicate that contrast-enhanced arterial-phase sequences may provide more informative biomarkers when used in univariate analyses, while T2-weighted features benefit more from integrative modeling approaches. This complementary behavior underscores the value of sequence selection based on the analytical context, with T1-weighted images offering stronger univariate predictors and T2-weighted images excelling in multivariate or deep learning frameworks.

These results are consistent with previous literature supporting the utility of radiomics for axillary lymph node (ALN) assessment in breast cancer.

Liu et al. [4] demonstrated the value of preoperative MRI in ALN detection, with radiomic features helping to improve diagnostic accuracy by quantifying tumor heterogeneity and architectural disruption within the nodes. For example, Song et al. [5] found that integrating radiomic features from DCE-MRI with clinical factors resulted in a predictive model with an AUC of 0.874, indicating the potential for combining imaging data and patient characteristics to enhance diagnostic accuracy.

Similarly, Gong et al. [11] performed a meta-analysis across 30 studies and concluded that radiomic models based on MRI features showed strong diagnostic performance, with an overall AUC of 0.90, sensitivity of 86%, and specificity of 79% in predicting lymph node metastasis.

Moreover, Shan et al. [17] demonstrated that integrating radiomic features with kinetic curve patterns from DCE-MRI could further improve the accuracy of ALN detection. Their combined model achieved an AUC of 0.91 in the training cohort and 0.86 in the validation cohort.

In our findings, features extracted from subtracted arterial-phase T1-weighted images performed better in univariate analysis, where individual descriptors such as GrayLevelVariance, Entropy, and JointAverage demonstrated fair to good discrimination (AUCs ranging from 0.712 to 0.787). This indicates that arterial phase imaging provides radiomic biomarkers with strong standalone predictive value, even outside of multivariate modeling. For example, wavelet.LLL.gldm.GrayLevelVariance alone achieved an AUC of 0.787. These results are consistent with previous studies [5, 11, 34], which have highlighted the additional diagnostic value of dynamic contrast-enhanced imaging in lymph node characterization. The strong performance of arterial-phase imaging, particularly through dynamic contrast enhancement, aligns with the work of Kim et al. [34], who emphasized the importance of early kinetic parameters in predicting breast cancer outcomes using DCE-MRI.

Overall, these results suggest a complementary role for both imaging sequences: arterial-phase T1-weighted features are highly informative when analyzed individually, while T2-weighted features gain greater diagnostic strength when exploited through machine learning and multivariate modeling. This supports the integration of both image types in future radiomics-based diagnostic strategies for ALN metastasis detection.

Feature importance analysis of the NN model further underscored the predictive role of texture-derived features such as LargeAreaLowGrayLevelEmphasis, DependenceVariance, and InverseVariance, particularly from wavelet-decomposed images. These metrics likely capture subtle spatial and intensity variations within the nodes, reflecting microstructural alterations associated with metastatic infiltration. Shape-based features like Elongation and Flatness and higher-order statistics such as Entropy also emerged as relevant contributors, suggesting that both geometric and textural heterogeneity are key to accurate classification.

While our study demonstrates the potential of radiomics in detecting metastatic ALN, it is not without limitations. First, the retrospective design of the study may introduce selection bias, as only patients who underwent MRI within two weeks before surgery were included. Additionally, our sample size, although sufficient for initial exploratory analysis, was relatively small compared to larger, multicenter studies, which may limit the generalizability of our findings. We added at the end of discussion section this sentence: Despite these limitations, the internal validation procedures yielded consistent and promising results: we have implemented robust statistical methodologies, including feature selection via Elastic Net regularization, tenfold cross-validation, and independent test set evaluation, which enhance the internal validity of our models. These procedures contribute to increasing the statistical power of the analysis and reducing the risk of overfitting. Consequently, the consistency of model performance across different machine learning approaches reinforces the reliability and robustness of the study results. Future work will focus on the inclusion of multicenter external cohorts to enhance the reproducibility and clinical applicability of the proposed radiomic models. Another limitation is that we focused exclusively on MRI sequences (T2-weighted and arterial-phase T1-weighted imaging) without integrating other imaging modalities like ultrasound or PET-CT, which may provide complementary diagnostic information [34–48].

## Conclusion

In conclusion, this study highlights the potential of MRI-based radiomic analysis, particularly when implemented through deep learning approaches such as neural networks, to serve as a noninvasive tool for the accurate detection of metastatic axillary lymph nodes in breast cancer patients. The strong diagnostic performance observed underscores the value of texture-based and morphological imaging biomarkers in clinical decision-making. Future research should focus on validating these findings across larger, multicenter populations and consider incorporating additional imaging modalities and clinical parameters to enhance the robustness and generalizability of radiomics-driven predictive models for lymph node metastasis.

## Data Availability

The datasets used and/or analysed during the current study are available at link https://zenodo.org/records/17048966.
